# Patients’ Opinions Toward Healthcare Quality and Improvement in Aseer Health: A Cross-Sectional Study

**DOI:** 10.7759/cureus.33432

**Published:** 2023-01-05

**Authors:** Abdullah Saeed, Abdullah AlShafea, Foton A, Tahani AlQadi, Abdulrahman Bin Saeed

**Affiliations:** 1 Action Research, Ministry of Health, Abha, SAU; 2 Research Unit, Ministry of Health, Abha, SAU; 3 Public Health, King Khalid University, Khamis Mushait, SAU; 4 Risk, Ministry of Health Holdings, Abha, SAU; 5 Public Health, King Abdelaziz University, Khamis Mushait, SAU

**Keywords:** public health care, • access to healthcare and health outcomes of vulnerable populations, chronic disease managment, population health management, quality indicator

## Abstract

Introduction

Population health is crucial to government planning. The 2030 national vision is transforming all Saudi sectors. The health sector revolution intends to boost life expectancy by reducing mortality due to road traffic accidents and morbidity due to chronic illness. Health services will change from treatment-based to person-centered and preventive care, empower the population, increase access to healthcare and improve service quality by offering high customer satisfaction. This study establishes a baseline assessment for public awareness, behavior, healthcare access, and quality.

Method

A cross-sectional study was conducted in the Aseer region in southwestern Saudi Arabia in 2022, September and October. Using the Aseer region's 2.1 million people to calculate the sample needed, the minimal sample size was 664. The study used cluster random sampling and a structured self-administered questionnaire to meet health transformation strategy requirements. All study variables underwent descriptive and bivariate analysis.

Result

The survey received 1,381 responses, with 97.2% of participants being Saudi and 84.4% being male. 312 (22.6%) of participants self-reported their health status as weak; 615 (44.5%) self-reported as having a good health status; and 667 (48.3%) reported having high knowledge. 740 people (53.6%) said that health was very important in their daily lives. The evaluation rate for Ministry of Health services in primary healthcare centers was 585 (42.4%) medium and 398 (28.8%) media for inpatient services. Evaluation for surgical procedure availability was 388 (28.1%) media. The results showed there is a statistically significant relationship (p-value < 0.05) between health status and age, gender, knowledge, and the importance of a healthy lifestyle. And there is a statistically significant relationship between the availability and quality of the health service profile, including inpatient, outpatient, and virtual care. Nationality appears to be statistically insignificant.

Conclusion

Health promotion is effective because people are health conscious. The importance of health and lifestyle presents a tremendous opportunity to incorporate health into all policies and improve the availability of healthy lifestyle options and surroundings to support healthy behaviors that will reduce preventable disease and risk mortality and morbidity. Health status was also linked to healthcare availability and quality.

## Introduction

The health of the population is a critically important aspect of government planning. The Kingdom of Saudi Arabia adopted “Vision 2030” as a strategic plan for developing its economy and national growth, and subsequently, the national transformation program (NTP) was established. A new model of care (MOC) was designed as a part of transformational healthcare, which is the first of the eight themes of the NTP. This new MOC plans to reform the healthcare system, aiming to make it more comprehensive and beneficial. It will deliver 42 coordinated interventions within six systems of care (SOC) by the end of 2020. All sectors in Saudi Arabia are undergoing a transformation in accordance with the national vision 2030 [1]. Road traffic accidents are the main cause of injuries in Saudi Arabia, accounting for 52% of all injuries. This amount is five times greater than the 12% average for the entire world. According to the most recent WHO data, 12,317 people died in road traffic accidents in Saudi Arabia in 2020, accounting for 9.19% of all fatalities. Also, the prevalence of chronic diseases is rising and accounted for about 60% of all premature deaths in 2016. This transformation in the health sector has a plan that aims to increase the life expectancy of the population by decreasing the risk of injury from road traffic accidents and decreasing mortality due to chronic diseases. The delivery of health services will shift from treatment-based and patient-centered to person-centered care, shift toward preventive care, and empower the population, increasing access to healthcare and service quality by providing a high level of customer satisfaction [2].

A number of measures for health status have been developed that can be used to compare various sociodemographic groups, evaluate the effectiveness of healthcare interventions, and measure population health in general. These measures focus on the degree to which people's abilities to function physically, socially, and mentally are compromised, going beyond conventional indices of mortality or morbidity [3]. Life expectancy, child deaths, major death causes, adjusted life years with disability, the number of disability-adjusted life years, and their causes were evaluated in Kazakhstan. Life expectancy fell between 1990 and 1996 (69-71 years) but then recovered, and mortality among children under the age of five decreased. The major death causes were noted to remain ischemic heart diseases, stroke, and chronic obstructive pulmonary diseases, while cardiovascular risk factors were the largest cause of disability [4]. In western China, rural citizens' perceptions of health concerns were fairly low, and the ways of learning about their health concerns were simple and ordinary [5].

These systems are: Keep Well; Safe Birth; Planned Care; Urgent Care; Chronic Conditions; and Last Phase [6]. New technologies, such as high-speed Internet, video conferencing tools, and digital examination tools, have enabled in-person visits to be replaced with aided virtual ones [7]. In the context of tightened budgets, increasing costs, and fundamental changes in the organizational infrastructure of health care, telemedicine is emerging rapidly [8]. Obviously, COVID-19 has exhibited many benefits of telehealth; therefore, it should persist as a permanent way of healthcare delivery, keeping in mind its future developments [9]. There is a local study explaining the challenges that face a healthy lifestyle done in Riyadh, Saudi Arabia, concluded that the biggest obstacle to physical activity was a lack of resources, while adherence to physical activity and a healthy diet were avoided due to a lack of willpower and social support [10]. In another study, a Saudi sample revealed that younger men and older women are at higher risk of an unhealthy lifestyle. In addition to self-motivation, combined strategies to promote physical activity and healthy eating are required to improve lifestyle [11].

In 2018, there were 75,225 beds in 484 hospitals in Saudi Arabia, with an average of 22.5 beds per 10,000 population, and the total budget for the health sector reached 90 billion SR (9.2% of the total governmental budget) [12]. Patient satisfaction is a major index for measuring healthcare quality and is associated with clinical outcomes [13]. It is important for many causes. First, patients who are satisfied with the care they receive tend to keep good relationships with the providers and respond to instructions and follow advice. Second, by understanding the causes of patient satisfaction and dissatisfaction, providers can address their strong points and continue, as well as their weaknesses and try to improve them. Third, the measurements used to determine patient satisfaction add valuable information about the performance, thus contributing to the total quality. Healthcare infrastructure is appropriate in Saudi Arabia regarding facilities and staff; improving staff performance and patient happiness is the real issue [14]. In a study done in the surgical ward at King Abdulaziz University Hospital in Jeddah, Saudi Arabia, the overall satisfaction rate was 89.6%. Age (greater than 50 years) and male gender were found to be significantly correlated with patient dissatisfaction, which can provide information about the patient groups that need extra attention from the hospital throughout their management. The length of the hospital stay is a significant contributing factor to patient unhappiness [15]. There is the use of technology in healthcare that can improve access and decrease waiting lists in surgical services and improve the average length of stay. Many studies have shown that using new surgical techniques like laparoscopic surgery, single port surgery, robotic surgery, outpatient surgery, and day surgery can lead to quicker patient recovery periods and shorter hospital stays, both of which will increase patient satisfaction [16,17].

The Ministry of Health in Saudi Arabia launched Ada’a Health in 2017. It is a program for performance management of healthcare services that aims to build a foundation for achieving the health goals of the Saudi Vision 2030. Many key performance indicators (KPIs) have been put in place. They include KPIs for the emergency department (ED), which are composed of Door to Doctor, Doctor to Decision, Decision to Disposition, and Percentage of Non-Urgent Patients. The benchmarks were as follows: Door to Doctor: time from registration to time seen by a physician, calculated as a percentage of the total time spent in the ER. Benchmark: world-class: 10%; acceptable: 20%; needing improvement: 40%; and unacceptable: 50%. Doctor to Decision: time from being seen by the physician to the time of the decision (admit, refer, or discharge). World-class time: 30 minutes; acceptable time: 59 minutes; needing improvement: 85 minutes; unacceptable time: 120 minutes. The decision Disposition: from the time of the decision to the time of leaving the ED Benchmark: word class: 30 minutes; acceptable: 90 minutes; needing improvement: 130 minutes; unacceptable: 131 minutes. Non-Urgent Patients: the proportion of patients triaged at level 4 or 5. Benchmark: world-class: 33%; acceptable: 50%; needing improvement: 75%; unacceptable: 95% [18]. The aim of this study is to establish a baseline assessment of population awareness, behavior, access to healthcare, and the quality of service provided. Population health can be defined as the health outcomes of a group of people, including the distribution of these outcomes within the group.

## Materials and methods

Study design

This study employed a cross-sectional descriptive research design because it provides quantitative evidence about healthcare quality improvement in Aseer Health through data collection, analyzing, and reporting. Therefore, a quantitative cross-sectional survey was conducted to explore patients' attitudes toward safety using a self-administered questionnaire. The self-administered questionnaire approach was adopted because it made it easy to reach a large number of participants within a short time. Thus, it eliminates interviewer bias. Again, this study acquired a descriptive design to acquire information about the current state of healthcare in Aseer Health and describe the relationship between variables.

Setting

The study was conducted in a natural setting through electronically collected. The included facilities were primary care centers, government hospitals affiliated with the Ministry of Health, and for-profit private hospitals. The recruitment sources were outpatient clinics and tertiary care centers from government hospitals, for-profit private hospitals, and primary care centers in the Aseer region.

Data was collected using closed-end questionnaires that captured the various variables of the study. This data collection method was selected because it allowed for the collection of subjective and objective opinions in a large sample of the population to obtain statistically significant results. Questionnaires are also good tools for protecting the privacy of the participants. Before the required data was collected, official approval was obtained from Central Institutional Review Board with number IRB 22-17-E. Participants consented to the study after being informed of its purpose. All questionnaires were anonymous and collected no personal data to protect data privacy. Questionnaires were distributed in hard copies and electronic formats to those patients who could not be reached easily. 1381 questionnaires were collected over two months (September-October 2022). The follow-up took place two months after the study was conducted.

Participants 

The study participants were patients who had received treatment from the healthcare facilities in the Aseer region. A total of 1,500 participants were selected as eligible to participate in the study in the selected setting. The total number of responses was 1,381 with a 92% response rate. A cluster sampling technique in the Aseer region divided into four clusters based on geographic location was used to select eligible participants since it was not feasible to determine the sampling frame in all hospitals in the Aseer region. To meet eligibility criteria, the participants were supposed to have attained the age of 18 years and above. Also, both male 1,165 (84.4%) and female 216 (15.6%) participants took part in the study. They were supposed to have received medical treatment in the Aseer region or undergoing medication at the time of the study.

Variables

**Figure 1:**
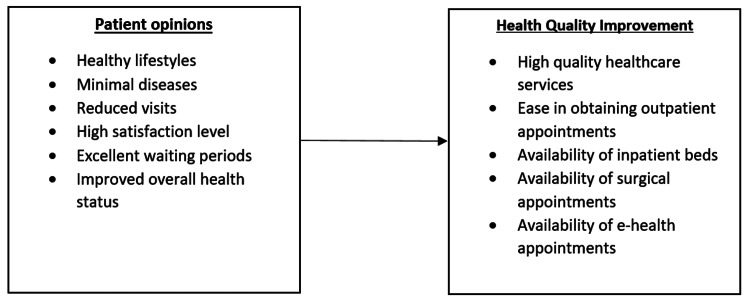
The variables of the study

The variables are the phenomena that the research tries to measure. They include dependent and independent variables. The dependent and independent variables are patient opinions and health quality improvement, respectively. The dependent and independent variables are further subdivided as shown in Figure 1.

Data sources/measurement

The opinion of patients Towards Healthcare Quality and Improvement was measured using a 17-item questionnaire containing questions about the patient's demographic characteristics and questions related to healthcare quality improvement. The responses were then calculated on a four-point Likert scale with items; “excellent,” “average,” “weak” and “I do not know.” Excellent meant a higher level of knowledge and a positive attitude of patients toward healthcare quality improvement, while “I don’t know” meant a lack of knowledge about the study questions.

Bias

To address potential sources of inherent bias, the study tools were translated into Arabic and English to suit the culture of different people in the Aseer region. Also, to avoid participant bias, steps were taken to guarantee the respondents of their anonymity when answering questions presented in the questionnaire. An internal consistency technique was also applied to assess the instruments' reliability. To avoid selection bias, everyone in the population had an equal chance of being selected to participate in the study. In this case, randomization was applied.

Study size

A sample size is the section of the main population selected to conduct a study. A convenience sample from cluster of patients who had visited government hospitals, for-profit private hospitals, and primary care centers in the Aseer region was selected to participate in the study. The population of patients eligible to take part in the study was 1,500. To calculate the sample size, the confidence interval was determined to be 99%. Using the sample size converter, a population (N) of 1,500 participants, a confidence level of 99%, and a 0.5% standard deviation gave a sample size (n) of 664 participants.

Quantitative variables

In the study, quantitative data in the questionnaire related to the age of participants, the number of visits the participants have made to hospitals in their area, and the number of visits made to primary care centers. These variables are expressed in numbers. Therefore, overall scale scores were used to analyze the collected data by setting the statistical significance point at p ≤ 0.05. Quantitative data were also analyzed using descriptive statistics (frequency and percentage) by determining the number of times the values were observed in the scale.

Statistical methods

Data analysis means bringing structure and meaning to a large volume of data. Data gathered from questionnaires were scrutinized thoroughly and carefully to ensure that the final data for coding was complete, accurate, and relevant. This was achieved by counter-checking all the responses in the questionnaires to discard any incorrect responses. Qualitative data were analyzed by gathering, summarizing, and categorizing it, then presenting it in narrative form. Quantitative data were analyzed through inferential statistics by chi-square. The tool for analysis was the statistical Package for Social Science (SPSS) installed on a personal computer. We used statistically significant level (a = 0.05), with CI 95%.

## Results

In Table 1, the survey received 1,381 responses, with 97.2% of participants being Saudi and 84.4% being male. 312 (22.6%) of participants self-reported their health status as weak; 289 (20.9%) self-reported their health status as moderate; and 615 (44.5%) self-reported having a good health status. Only 66 people (4.8%) reported having low knowledge about ways to stay healthy, while 618 (44.8%) reported having moderate knowledge, and 667 (48.3%) reported having high knowledge.

**Table 1:** Descriptive analysis of all study variables

Variable	Frequency	Percentage
Age category		
Above 60	123	8.9%
51-60	262	19%
41-50	401	29%
31-40	397	28.7%
20-30	198	14.3%
Gender		
Male	1165	84.4%
Female	216	15.6%
Nationality		
Saudi	1343	97.2%
Non-Saudi	38	2.8%
Place of resident		
Abha	521	37.7%
Muhayl	442	32%
Khamis Mushayt	144	10.3%
Other	282	20%
Health status currently (Self report describe)		
Weak (I have chronic disease)	312	22.6%
I don't know	165	11.9%
Moderate (I have one or more risk factor such as smoking, high weight)	289	20.9%
Good (I have healthy lifestyle)	615	44.5%
Knowledge about ways to stay healthy		
I don't know	30	2.2%
Low knowledge	66	4.8%
Moderate	618	44.8%
High	667	48.3%
The level of importance to your health in daily lifestyle		
I don't know	15	1.1%
Low importance	81	5.9%
Moderate	545	39.5%
High	740	53.6%
Chronic disease prevalence (self-reporting)		
Diabetes mellitus	277	20%
Hypertension	243	17.6%
Obesity	142	10.3%
Asthma	91	6.6%
Epilepsy	82	6%
No chronic disease	546	39.5%
The opinion about factors have negative impact on lifestyle		
Time management	521	37.7%
Lack of awareness	218	15.7%
Difficulty to access to healthcare	181	13.1%
Lack of activity in school and work	311	22.5%
Lack of supportive environment such as walk area	159	11.5%
Number of user of facility other than Ministry of Health		
Outside Aseer region	81	5.8%
Armed force hospital	173	12.5%
Private	420	30.4%
Ministry of Health	674	48.8%
Evaluation of service in Ministry of Health in hospital		
I don't know	13	1%
Weak	510	36.9%
Medium	611	44.2%
High	247	17.9%
Evaluation of service in Ministry of Health in primary healthcare		
I don't know	44	3.2%
Weak	513	37.1%
Medium	585	42.4%
High	239	17.3%
Evaluation of finding appointment and availability		
I don't know	42	3%
Weak	737	53.4%
Medium	438	31.7%
High	164	11.9%
Evaluation of finding beds and inpatient service		
I don't know	133	9.6%
Weak	747	54.1%
Medium	398	28.8%
High	103	7.5%
Evaluation of availability of surgical procedure		
I don't know	182	13.2%
Weak	708	51.3%
Medium	388	28.1%
High	103	7.5%
Evaluation of waiting time in emergency department		
I don't know	47	3.4%
Weak	923	66.8%
Medium	335	24.3%
High	76	5.5%
Evaluation of private hospital service		
I don't know	87	6.3%
Weak	384	27.8%
Medium	695	50.3%
High	215	15.6%
Evaluation of electronic and virtual service		
I don't know	58	4.2%
Weak	216	15.6%
Medium	548	39.7%
High	559	40.5%
Priority of health service in region from your opinion		
Improve the primary care service	417	30%
Improve virtual care	151	11%
Chronic disease service	628	45.4%
Women health	185	13.6%

When asked about the level of importance of health to their daily lifestyle, 81 (5.9%) of participants reported that health had low importance to their daily lifestyle, 545 (39.5%) reported it to have moderate importance, and 740 (53.6%) reported it to have high importance. The most common chronic illness was diabetes mellitus, which was reported by 277 people, or 20%. 243 people, or 17.6%, reported having hypertension. Obesity was reported by 142 people (10.3%), 91 people (6.6%), and 82 people (6%).

When participants were asked to name things that hurt their lifestyle, 521 (37.7%) said poor time management; 311 (22.5%), a lack of activity; 218 (15.7%), a lack of awareness; 181 (13.1%), trouble getting medical care; and 159 (11.5%), a lack of a supportive environment like a walking area. The number of people who use facilities other than those run by the Ministry of Health is 420 (30.4%) for private facilities, 173 (12.5%) for the Armed Forces Hospital, and 81 (5.8%) for places outside the Aseer region, while the number of people who use Ministry of Health facilities is 674 (48.8%).

When asked to evaluate the services of the Ministry of Health in hospitals, 510 (36.6%) reported them as weak, 611 (44.2%) reported them as medium, and 247 (17.9%) reported them as good. while evaluations for Ministry of Health services in primary healthcare were reported as 513 (37.1%) weak, 585 (42.4%) medium, and 239 (17.3%) good. When asked to evaluate the difficulty of finding appointments and availability, 737 (53.4%) answered weak, 438 (31.7%) medium, and 164 (11.9%) high. As for finding beds and inpatient services, 747 (54.1%) answered weak, 398 (28.8%) medium, and 103 (7.5%) high.

For the evolution of the availability of surgical procedures, 708 (51.3%) answered weak, 388 (28.1%) medium, and 103 (7.5%) high. While the evolution of waiting times in the emergency department is 923 (66.8%) weak, 335 (24.3%) medium, and 76 (5.5%) high, the evolution of private hospital services is 384 (27.8%) weak, 695 (50.3%) medium, and 215 (15.6%) high.

When asked how they felt about electronic and virtual services, 216 people (15.6%) said they were weak, 548 said they were medium, and 559 said they were high. Participants were asked about the priority of health services in the region according to their opinion; 628 (45.4%) answered with chronic disease services, 417 (30%) improved primary care services, 185 (13.6%) improved women's health, and 151 (11.1%) improved virtual care.

The significant differences were tested using chi-square in Table 2, with the dependent variable being current health status, as well as all of the other variables described in Table 2. The results of the Chi-square test show there is a statistically significant relationship between health status and age, gender, knowledge, and the importance of a healthy lifestyle. And there is a statistically significant relationship with the availability and quality of the health service profile, including inpatient, outpatient, and virtual care. The nationality appears to be insignificant statistically.

**Table 2:** Bivariate analysis of study variable *Health status currently is the variable that test with all variable in the table. **Not statically signifacnt.

Variable	Test value (Chi-square)	P.value
Age category	109.7	0.000
Gender	9.6	0.022
Nationality	0.487	0.922**
Knowledge about ways to stay healthy	102.1	0.000
The level of importance to your health in daily lifestyle	100.2	0.000
Chronic disease prevalence (self-reporting)	144.1	0.000
The opinion about factors have negative impact on lifestyle	107.2	0.016
Number of user of facility other than Ministry of Health	162.5	0.002
Evaluation of service in Ministry of Health in hospital	37.5	0.000
Evaluation of service in Ministry of Health in primary healthcare	24.9	0.003
Evaluation of finding appointment and availability	33.3	0.000
Evaluation of finding beds and inpatient service	47.5	0.000
Evaluation of availability of surgical procedure	59.5	0.000
Evaluation of waiting time in emergency department	43.7	0.000
Evaluation of private hospital service	20.8	0.014
Evaluation of electronic and virtual service	45.9	0.000

## Discussion

The majority of the participants were Saudi men (84.4%) between the ages of 31 and 50 (57.7%). Residents Abha and Khamis Mushait, representing the high-altitude area, and Muhayl, representing the low-altitude area, had the most disturbance of place. The other represents the region's remaining rural areas. The overall health was good, and 20% of people had one or more risk factors; this necessitates more effort in raising awareness and promoting health, and interventions can correct the lifestyle. Participants reported a level of knowledge and importance about health that was greater than 90% for moderate-to-high knowledge.

Comparing our study with the study, which examined a sample population in Dammam to determine the prevalence of hypertension at 13.5%, [19] While our result was 17.6%, this may be related to different genetic factors and geographical differences. The highest prevalence was found in the 40-year-old age group (15.3%) [20]. The explanation for this result being higher than the national rate is due to an increased family history of diabetes and an unhealthy lifestyle, which lead to an increase in the risk factor. The region's asthma rate was 6.6%, which was slightly higher than the national rate of 4.1% [21].

The goal of Saudi Vision 2030 and the Saudi Health Transformation Program is to improve healthcare access and quality of service, increase expected life expectancy by lowering major risk factors, and improve quality of life by promoting and making healthy choices more accessible in order to increase healthy lifestyle adherence. This will decrease the prevalence of chronic disease and decrease the need for service, and the improvements in virtual care will decrease the number of actual visits. When you add up all of these things, they can lead to shorter wait times in the emergency room, the outpatient department, and the inpatient department.

Strength and limitations

The strength of the study was the adequate sample size and random distribution through the region zone which make it representative of the population. The limitation was using a self-reporting questionnaire could lead to self-reporting bias, and the study is mostly male because it was hard to get access to the female community. This could mean that women's health problems are not taken into account as much as they should be, so we suggest that future studies focus on the female population. The quality assessment did not use a good questionnaire, and the report was just a list of raw variables. This was seen as a knowledge gap that needed to be filled by a new study.

## Conclusions

The population has a high level of awareness and knowledge about health, which supports the effectiveness of the role of health promotion. The importance of health and lifestyle reflects a great opportunity to incorporate health into all policies and improve the availability of healthy choices and environments to encourage the population to adopt healthy behaviors that will reduce mortality and morbidity from preventable disease and risk. Furthermore, the study found a link between health status and the availability and quality of healthcare services.

The Aseer region has a large geographic area with low and high altitudes, and the major issues it faces in healthcare are increasing demand for services while decreasing access and increasing waiting times in the emergency department, scheduling appointments for outpatient care, obtaining a surgical procedure schedule, and a high bed occupancy rate. The application of a health transformation plan by shifting to person-centered care and improving prevention and quality of life with increased virtual service can lead to improvements in all these factors.

Recommendations 

Because they lack a primary care physician and recommended health services, such as cancer screenings. Other times, it is because the healthcare professionals who offer services are too far away from where they live. More people can receive the care they require with the aid of initiatives to improve communication in-person or remotely between healthcare providers and patients. A patient has access to care if they can see a qualified healthcare professional in a timely manner.

This can be accomplished by integrating modern technologies into hospitals, such as electronic health records. Improved training for nurses and doctors on how to avoid mistakes that could result in damage or death is another option to raise the standard of patient care.
